# Successes and obstacles in implementing social health insurance in developing and middle-income countries: A scoping review of 5-year recent literatures

**DOI:** 10.3389/fpubh.2022.918188

**Published:** 2022-10-26

**Authors:** Mohammad Husni Jamal, Aznida Firzah Abdul Aziz, Azimatun Noor Aizuddin, Syed Mohamed Aljunid

**Affiliations:** ^1^University of Cyberjaya, Cyberjaya, Malaysia; ^2^Academy of Family Physicians of Malaysia, Kuala Lumpur, Malaysia; ^3^Department of Family Medicine, Faculty of Medicine, Universiti Kebangsaan Malaysia, Cheras, Malaysia; ^4^Department of Community Health, Faculty of Medicine, Universiti Kebangsaan Malaysia, Cheras, Malaysia; ^5^International Centre for Casemix and Clinical Coding, Hospital Canselor Tuanku Muhriz, Universiti Kebangsaan Malaysia, Cheras, Malaysia; ^6^Department of Health Policy and Management, College of Public Health, Kuwait University, Kuwait City, Kuwait

**Keywords:** social health insurance, community health insurance, national health insurance, successes and obstacles, developing countries, low and middle income countries

## Abstract

Social health insurance (SHI) is a form of health finance mechanism that had been implemented in many countries to achieve universal health care (UHC). To emulate the successes of SHI in many developed countries, many developing and middle-income countries (MICs) have attempted to follow suit. However, the SHI implementation has problems and obstacles. Many more obstacles were observed despite some successes. This scoping review aimed to study the various developments of SHI globally in its uses, implementation, successes, and obstacles within the last 5 years from 2017 to 2021. Using three databases (i.e., PubMed, EBSCO, and Google Scholar), we reviewed all forms of articles on SHI, including gray literature. The PRISMA-ScR protocol was adapted as the guideline. We used the following search terms: social health insurance, national health insurance, and community health insurance. A total of 57,686 articles were screened, and subsequently, 46 articles were included in the final review. Results showed that the majority of SHI studies were in China and African countries, both of which were actively pursuing SHI programs to achieve UHC. China was still regarded as a developing country. There were also recent experiences from other Asian countries, but only a few from South America. Implementing SHI to achieve UHC was desirable but will need to consider several factors and issues. This was especially the case in developing and MICs. Eventually, full UHC would only be possible with a combination of general taxation and SHI.

## Introduction

Social health insurance (SHI) was a form of health finance mechanism to increase the efficiency of healthcare systems. This concept had been successfully implemented in many developed countries and had contributed to achieving universal coverage and overcoming equity issues. Developing and middle-income countries (MICs) have attempted to implement such schemes with varying outcomes.

By definition, SHI had three distinct characteristics. First, enrolment was compulsory, and participants must pay the specified premium or contribution. However, in practice, contributions may initially be voluntary. Second, only registrants were entitled to the benefits when premiums had been paid. Finally, SHI involved legislation that documented the benefit packages entitled to the participants for the amount of premium paid. The selected SHI articles included in this article involved wide variations of SHI practices. However, all of them embodied the basic functions of SHI as a form of health finance mechanism that involved revenue collection, pooling of risks, and purchasing. The two important health finance mechanisms to achieve universal healthcare (UHC) were general taxation and SHI. This review aims to gauge the effectiveness of SHI in achieving UHC with or without the complementary engagement of general taxation.

The World Bank classified each of the world's nations into one of four categories: high, upper-middle, lower-middle, and low income. Upper-middle and lower-middle income countries were known collectively as middle-income countries (MICs). To accommodate shifts in the global economy, the World Bank adjusted the boundary lines between the categories every July. For 2021–2022, lower-middle-income countries are those with a gross national income (GNI) per capita of $1,046–$4,095. Meanwhile, upper-middle-income countries have a GNI per capita of $4,096–$12,695.

**Figure 1:**
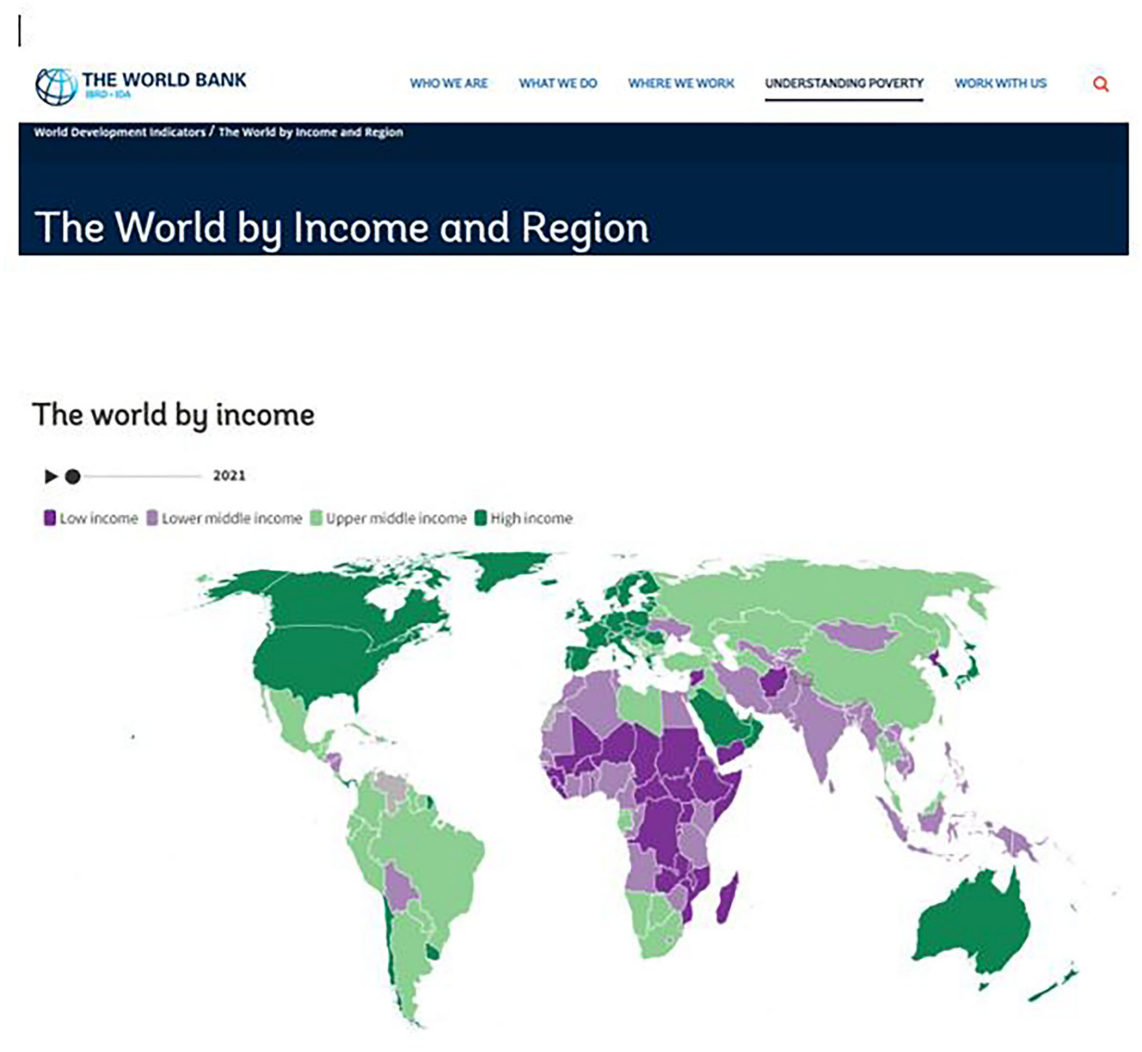
World by income (source: The World Bank).

The United Nations assessment of the Human Development Index categorized middle-income countries as developing countries. In particular, developing countries rank above least-developed countries (which typically correlate to low-income countries) but fall short of developed (high-income) countries (https://worldpopulationreview.com/country-rankings/middle-income-countries). Figure 1 presents the World Bank classification of the countries of the world based on income. The actual figure in the article is interactive, indicating the actual category of each country when clicked on this link (https://datatopics.worldbank.org/world-development-indicators/the-world-by-income-and-region.html). Note from this classification that Rwanda was classified as a low-income country whereas Chile and Uruguay were classified as high-income. However, these three countries were part of a series of multi-country studies, wherein the vast majority were in the middle-income category from articles related to this study.

Additionally, China, which was another country in this study, was still considered a “developing country” based on the criteria of the World Bank and the United Nations. This classification was based on the following indices as reported. First, in 2019, China's per capita GNI was only $10,410, which made it an upper-middle-income country, as defined by the World Bank, but not a high-income country. The country had a human development index (HDI) of 0.761, putting it in the high human development tier (developed countries have quite a high human development). China's uneven distribution of wealth was another factor. This is evidenced by much of its economic growth being concentrated in coastal areas rather than in the interior provinces. Approximately 60% of China's population lived in urban areas. However, the average in developed countries maintained around 80%. Additionally, a wide wealth gap exists between those who lived in China's cities and those who continued living in rural areas (Is China A Developing Country? WorldAtlas; https://www.worldatlas.com > Jason Shvili, 18 January 2021, in World Facts).

“One of the first countries that instituted SHI nationally was Germany in 1883. Since then, the concept of SHI reached throughout the world.” SHI comprises structural components. These components include the establishment *via* government legislation; regular and compulsory financial contributions; the absence of any possibility for enrollees to opt-out of a scheme; the premium rates based on the ability to pay, and, finally, standardized benefit packages. Additionally, handling risk adjustments would be an important function. Payment models could be in the form of a single or multi-payer system. The advantages of a single-payer system were ease of revenue collection, efficiency, overall cost control, and subsidy coverage for the poor (1).

Important considerations in SHI implementation planning included per capita income, capacities of formal and informal sectors, geographical population distribution such as urban or rural locations, SHI structure (multiple or single, voluntary or compulsory), and target groups (e.g., elderly, employees, and self-employed) (1).

Supervision was an important element in SHI to ensure that the various funding bodies must act in accordance with the regulations stipulated by the SHI, and tasks are related to the information collection, assessment, and intervention. Supervision ensured legitimacy, trust and stability, and efficiency and assisted in policymaking (2).

While having a sizable formal sector was a prerequisite for easy SHI implementation, some barriers to the successful outcome may still exist. These included the willingness and practicability of employers' contributions, employee resistance to making contributions, transparency concerns in the national insurance system, the function of states in federal SHI programs, and roles of policy advocates such as state governors (3).

Despite mandatory enrolment in the formal sector, private firms may not comply but offered better health packages for their employees. Employees were also not well informed by their employers regarding SHI benefits. Low-quality health services at public hospitals under SHI further contributed to this lack of preference for SHI (4).

The integration of informal sectors (population who were self-employed or with non-regular incomes) and coverage of the poor into an SHI scheme posed many problems as well. A lack of knowledge of SHI and its enrolment processes was the most critical barrier (5). However, some others had successful innovative methods, such as special low-income schemes especially focused on the poor and other vulnerable groups (6).

An important aspect of SHI was to recognize consumers' preferences in the selection of various health packages. In particular, a varied choice of health plans would lead to higher quality and cost-effective services at lower costs (7).

SHI and general tax financing were two sources of health financing. However, comparatively, SHI had several weaknesses. These included the area of revenue collection, whereby the dependency on formal sectors was greatly reduced with the presence of large informal sectors. In risk pooling, large informal sectors would take a longer time to achieve universal health coverage. Even with formal sectors, the coverage may not reach 100%. Multiple risk pools in SHI resulted in different benefits packages and hence various contribution payments, unlike general taxation. Regarding the purchase and provision of healthcare, SHI may not offer any added advantage in a purchaser-provider split and strategic purchasing, as general tax financing is equally capable of its achievement (8).

Another issue to consider was the “willingness-to-pay” for the premiums. This was an important prerequisite to understanding the citizens' level of acceptance before its implementation and the amount that they are willing to pay for premiums (9).

Factors that favor the successful implementation of SHI in a country included good economic development, a strong financial and administrative capacity of the government, and higher trade union density. Further, taxation agencies were better at collecting SHI premiums (10).

In addition to coverage for secondary and tertiary care, SHI can include benefit packages such as health information, education, counseling, and disease prevention, for primary care related to the prevention of non-communicable diseases (11).

Another important pitfall to avoid was the equity issue in SHI whereby the structure has only one standard for all, despite the income capabilities. This may benefit the rich more than the poor. The fault was due to the one-size-fits-all SHI structure. To achieve social justice, restructuring could be in the form of increasing the premium or decreasing the reimbursement tariff of the high-income group or vice versa for the low-income group (12).

Creating a national SHI scheme could be initiated by integrating various existing community-based healthcare financing schemes into the national SHI. One such example is PhilHealth in the Philippines. Benefits were classified into ordinary, intensive, and catastrophic packages, which encompassed a wide range of coverage and reimbursement mechanisms. Despite some shortfalls, PhilHealth had been successful in many areas and could be an example for other developing countries to emulate (13).

This scoping review aimed to answer the following questions: What were the latest developments in SHI practices in various countries? How many of these had successfully achieved UHC? What were the specific issues of concern?

## Methods

### Data sources and search strategy

A search *via* PubMed and Google Scholar noted that the current topic under study had not yet been attempted thus far. Three databases (i.e., PubMed, EBSCO, and Google Scholar) were searched between May and July 2021 for articles related to SHI. We used the following search terms: social health insurance, national health insurance, and community health insurance. The publication language was restricted to English, and articles were restricted from 2017 to 2021. We supplemented our review through a manual search of gray literature related to working articles. A detailed search strategy is presented in Table 1.

**Table 1:** Search strategy.

**Data-base**	**Keywords**
PubMed	Steps: 1. Select each term: social health insurance/national health insurance/community health insurance 2. Select full text 3. Select search by year from 2017 to 2021
Google Scholar	Steps: 1. Select each term: social health insurance/national health insurance/community health insurance 2. Select custom range: sort by date 2017 to 2021
EBSCO Medline (Discovery)	Steps: 1. Select each term: social health insurance/national health insurance/community health insurance (basic search) 2. Select subject; health insurance 3. Select full text 4. Sort range by dates: 2017 to 2021

### Study selection

The titles and abstracts of the documents were assessed against the inclusion and exclusion criteria.

a. The inclusion criteria cover all articles on SHI, including gray literature within a 5-year period from 2017 to 2021.
b. The exclusion criteria referred to all non-English publications.

References were managed using Mendeley Desktop and Mendeley Reference Manager.

### Data extraction and synthesis

The various studies were divided per the search strategy (Table 1), their country location (Table 2), and the nature of articles (Supplementary Table 3).

**Table 2:** Location/setting of articles by countries.

	**Region**	**Country**	**No. of articles**
1.	Asia	China	13
		Vietnam	3
		India	2
		Indonesia	2
		Philippines	2
		Iran	1
		Malaysia	1
		Mongolia	1
		Nepal	1
		Sri Lanka	1
2.	Africa	Nigeria	7
		Ghana	7 (2 in 2—country study; 2 in 5—country study)
		Kenya	6 (2 in 2—country study; 2 in 5—country study)
		Ethiopia	4 (2 in 5—country study)
		Rwanda	2 (2 in 5—country study)
		Tanzania	2 (2 in 5—country study)
3.	South America	Bolivia, Chile, Colombia, Costa Rica, Dominican Republic, Mexico, Peru and Uruguay	1

The Upper Mics are China, Colombia, Costa Rica, Dominican Republic, Malaysia, Mexico, and Peru.

The Lower Mics are Bolivia, Ethiopia, Ghana, India, Indonesia, Iran, Kenya, Mongolia, Nepal, Nigeria, The Philippines, Sri Lanka, Tanzania, and Vietnam.

Low income—Rwanda.

High-income—Chile and Uruguay.

Among the 46 selected studies, 27 were conducted in Asian countries, out of which 13 studies are from China, 18 studies from African countries, and 1 study from South American countries. And among the 46 selected studies, 28 were quantitative, 5 qualitative, and 3 mixed quantitative and qualitative, 2 narrative, 1 editorial, 1 commentary or viewpoint, 5 literature or document reviews, and 1 working article.

We observed the articles that fall under 11 broad themes and their numbers indicated: health finance mechanisms (7), universal health coverage (4), coverage of the poor (5), willingness-to-pay (6), healthcare service utilization (5), formal and informal sectors (4), strategic purchasing (3), internal migrants (3), enrolment (2), fund integration (2), and miscellaneous (5).

The main and corresponding authors were involved in the data abstraction.

We extracted the following key features from the studies included in the review: author and year, countries, objectives, type of study, population, and outcomes as represented in Supplementary Table 3.

## Results

**Figure 2:**
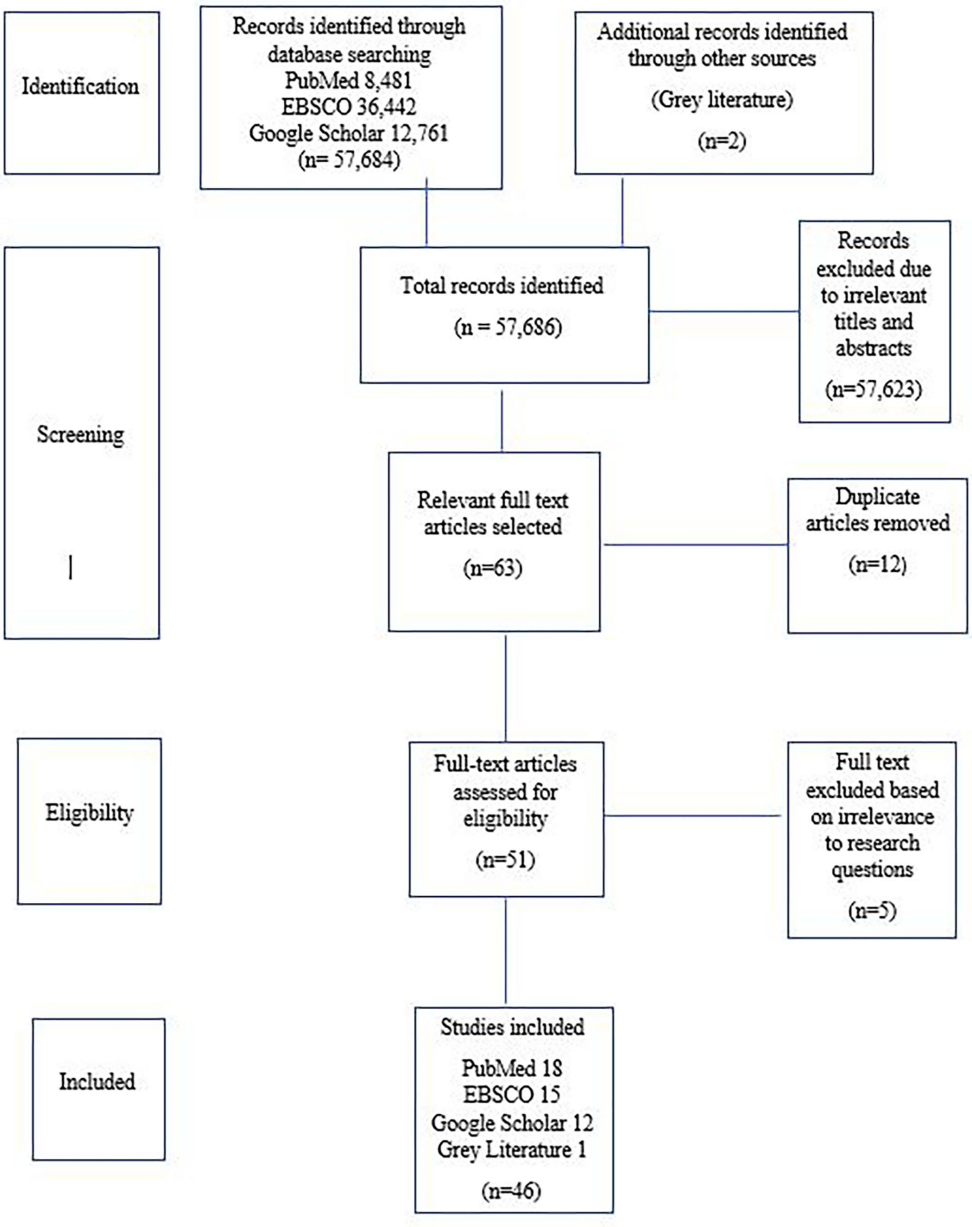
Prisma flow chart.

The results of the screening process are shown in Figure 2. A total of 57,686 documents were screened by title and abstract for possible inclusion in the review. After screening all the titles and abstracts, the full text of the selected documents was assessed against the eligibility and exclusion criteria, and 46 studies were selected and included in the final review.

### Summary by themes

#### Health finance mechanisms

In Nigeria, the four main health finance systems were general taxation, social health insurance, out-of-pocket, and donor funds. In the performance evaluation of each mechanism, the indicators included equity, efficiency, quality of services, and ability to prevent catastrophic health expenditure (CHE). For an effective function of the SHI, improvements to be introduced included compulsory enrolment into the national health insurance scheme (NHIS)/FSSHIP, coverage of informal sectors, prioritized strategic purchasing, and ensuring that enrollees were fully aware of their privileges. A sizable budget increase was required to accommodate the changes (14). Another Nigerian initiative was the development of a checklist to ensure a feasible and sustainable SHIS system scheme for middle-income countries. The final checklist had six domains focusing on finance sources, benefits packages, provider payment mechanism, contributing population and level of compulsion, pooling of funds, and finally, administration and management (15). A comparative assessment of financial requirements of both contributory (i.e., SHI) and non-contributory (i.e., general taxation) to finance UHC in Kenya with large informal sectors concluded that SHI was financially sustainable for 5 years but less beyond that. Meanwhile, the non-contributory (i.e., general taxation) model showed lower costs and more sustainability in both short and long term, especially for large informal sectors.

The non-contributory (general taxation) model had better long-term financial sustainability, but more innovation for general taxation methods is necessary (16). In China, another comparative analysis between SHI and private health insurance (PHI) *via* 3 channels, namely savings, demographic factors, and medical expenditure, concluded that SHI had a crowd-out effect in penetration, but a crowd-in effect in density. Therefore, the government should continue developing SHI and formulating incentive policies, such as preferential tax policy and moral hazard control measures (17). To assess the role of health maintenance organizations (HMOs) implementing national SHI in Enugu, Nigeria, the majority of respondents felt that HMOs did not meet their expectations and should improve in terms of accessibility, affordability, and equitability of distribution (18). The involvement of private healthcare providers in an SHI scheme should be made important as they contributed to the coverage of the underserved population. They should also be well informed regarding the need to be accredited, but the processes should not be burdensome. Incentives should be given to encourage their participation (19). SHI should reprioritize the coverage of outpatient services, especially for non-communicable chronic diseases, in place of inpatient services. This should be in the form of a higher reimbursement ratio for outpatient medical expenditures and would then encourage patients to seek more outpatient care (20).

Based on these experiences, the most likely workable solution was to create a hybrid scheme of general taxation with SHI, and factor in complementary or supplementary private insurance depending on the extent of coverage. Out-of-pocket contributions should be minimized, and the contribution of donor funds was only subject to availability and should not be an important integral of the structure.

#### Universal health coverage

UHC encompassed three important domains: population coverage, provision of benefits and services, and ensuring financial protection against out-of-pocket (OOP) and CHE. The Vietnam experience showed that, although SHI had contributed to UHC progress, dependence on voluntary enrolment had caused a shortfall in target. Additionally, prioritizing the population coverage and substituting generous packages with smaller benefits would allow more funds to be dispersed over a larger population (21). As part of its healthcare reform plan in 2009 to achieve UHC, China implemented three main types of SHI schemes—Basic Medical Insurance for Urban Employees (BMIUE), Basic Medical Insurance for Urban Residents (BMIUR), and New Rural Cooperative Medical Scheme (NCMS), and claimed to have achieved 95% UHC by 2011. However, the ground reality fell short as declared by SHI managers. Issues that required urgent resolution included increasing the funding capacity of SHIs and investment in healthcare. Inconsistent and disunited policies must be rectified, and effective and efficient fund management processes must be enforced (22). It was also observed in 2009 that the three SHI schemes accounted for <54% of the national healthcare expenditure. This is compounded by the problem of inappropriate unused surplus funds and ineffective payment methods. Creating a top-level reformatory design demanded expanding the coverage rate, increasing funding, providing better benefits, and modifying the payment method (23). To achieve UHC, the implementation of any SHI program must ensure that the structure must cover all groups, both formal and informal sectors, right from the beginning and not in sequential phases. The experiences of the five African countries had also proven that SHI alone was inadequate to ensure full UHC, but rather need the combination of tax-based funds to achieve the objective (24).

#### Coverage of the poor

SHI as a health finance mechanism should focus on three tenets of UHC which relate to population coverage, provision of benefits and services, and ensuring financial protection against OOP and CHE. The coverage must include all segments of society, including the poor and other vulnerable groups. The poor, who form the vulnerable segment of society, must not be required to pay for their healthcare and should be protected from any catastrophic expenses. However, the effectiveness of SHI in this respect was questionable as evidenced by a comparative study of the five African countries. Initially, the coverage of the poor was beset by problems of their specific categorization—any rigid definition would have exposed some to financial catastrophe. This was aggravated by the fragmentation of different SHI schemes and risk pools, thereby reducing cross-subsidies and sustainability of SHI schemes. Ensuring good governance with a strong political will help guarantee that the poor were not marginalized in SHI schemes (25). A similar situation was observed in the Philippines wherein the SHI scheme known as PhilHealth, despite providing access to the poor, failed to really achieve that target. The reasons for this were their lack of awareness due to insufficient education efforts on SHI benefits, physical remoteness from health facilities, and cultural tendency to opt for traditional forms of treatment. Remedial measures included promoting community-based care services, providing SHI information in simple languages along with health campaigns, and providing free medications (26). Failure to protect the poor was also observed in the Indian Rashtriya Swasthya Bima Yojana (RSBY) implemented in 2008. The scheme provided cashless health services to poor households in the form of a health card for hospital care. Therefore, no strong evidence that RSBY reduced per person OOP for poor households in both rural and urban areas (27). Another Indian SHI scheme in Karnataka displayed positive results in alleviating the poor. The SHI Vajpayee Arogyashree scheme (VAS) implemented in 2010, focused on increasing access to tertiary care. The VAS succeeded in improving healthcare for the poor and reducing their financial burden in tertiary care from covered conditions. The successes were attributed to the ease of use, community outreach activities, and coverage of high-burden disease conditions. Providers were selected from both the public and private sectors with bundled payments reimbursement, and access to services required pre-authorization (28). In an alternative attempt to assist the poor, a Chinese government grant in the form of cash known as medical financial assistance (MFA) was initiated to assist the poor in enrolment of the SHI schemes and to reduce their financial burden. The project failed on both counts in its forms. To overcome these issues, the Chinese government should instead invest more funds to ensure higher enrolment in SHI programs by the poor and to widen the benefits packages of MFA cash aid (29).

Effective coverage for the poor required a proactive approach that ensured ease of access, simple educational messages, broader benefits with a focus on high-burden diseases, and avoiding any OOP expenses.

#### Willingness to pay

The acceptability of the populace as regards their willingness to pay (WTP) for the premiums was an important factor to be considered before implementing any SHI programs. There have been positive responses in some countries, whereas there were negative responses in others. The Nigerian experience observed several socio-economic factors that influenced the WTP, such as household size, educational status, occupation, and household income. Despite 82% of the respondents supporting an average amount of 513 Naira (1.68 USD) per month per person was agreeable, only 65% were able to afford the premium. To address this, differences between the WTP and the cost of the benefits package should be identified, and the premium may need to be subsidized for the low-income group (30). Similarly, positive support was shown by government servants in Mekelle City, Ethiopia, wherein the survey revealed that 85.3% preferred social health insurance and were willing to pay for the scheme at 3.6% of their monthly salary. Meanwhile, this amount was lower in another group *via* the focus group discussions unless the quality of services was improved. Positive influencing factors included the level of income but were negatively associated with increasing age and educational status (31). In a more localized Nigerian study in the rural state of Akwa Ibom, a similar positive support of 82% were willing to pay, but only 30.1% were initially aware of SHI. Again, the support will depend strongly on the income level and family size (32). Meanwhile, the performance of the SHI scheme in Mongolia was disappointing, and only 40% were satisfied. This led to support for 1.7% of the average salary for the WTP for PHI. As a result, the following measures were proposed: expanding the SHI benefits, raising public awareness about SHI, and allowing PHI to complement SHI (33). Informal workers in Vietnam also expressed the WTP for 921.9 thousand Vietnamese dongs per household per year (US$42). This was influenced by the household income level, the health status of the household, and the number of uninsured members in a household. To encourage more enrolment of informal sector families, the premium should be affordable and subsidized by the government (34). SHI had yet to be implemented in Malaysia, but a local Sarawak study exhibited a negative response to it. Only 46.7% of the locals were supportive, but for only less than RM 20 monthly. Specifically, 53% did not agree to WTP, and from this number, 81.3% cited poor affordability. Almost half (48.1%) of the respondents were in the low-income group having less than RM 800 monthly income with an average family size of 3–5, and 47.7% had secondary education. Others suggested that it was the government's responsibility to provide healthcare. Hence, the priority was to increase their awareness on the benefits of SHI and to reduce any related misconceptions (35).

#### Healthcare service utilization

Depending on the structure of each SHI scheme, each would influence the behavior of enrolees on the usage of health facilities and the resultant costs. In a Chinese study with three different SHI schemes, it was found that the UEI and URI offer generous benefits packages, whereas NCM was the least generous. This resulted in UEI and URI schemes experiencing increased utilization of health services and total health costs. UEI obtained a high mean inpatient cost, and URI resulted in raised mean outpatient costs. Unfortunately, none of the three systems resulted in a significant reduction in the OOP expenses. A transformation from multi-payer to a single-payer function may result in increased healthcare utilization and costs if the SHI packages were to be generous. Therefore, cost control measures were required (36). Similar results in China were obtained in another more focused study involving the middle-aged and elderly. SHI had resulted in increased expenditure on healthcare costs due to increased utilization and also raised OOP costs. Hence, the government should adjust the three SHI schemes and optimize resource allocation to reduce inequality across the schemes (37).

In Nigeria, the views of enrolees and healthcare providers reflected that the SHI system called FSSHIP was not effective in providing universal financial risk protection (UFRP) and failed to guarantee UHC. The reforms proposed included enhancing the primary care gatekeeping to lower costs by preventing the bypass to more expensive secondary or tertiary care. The coverage of FSSHIP must also be widened, especially regarding inpatient services (38).

In Ghana and Kenya, SHI made it affordable for citizens to seek healthcare. However, many enrollees had to make co-payments because they were unaware of the accreditation status of the provider's full benefits of SHI. The coverage should expand access to primary healthcare, both to public and private providers, as there seemed to be more confidence in the private sector (39). Another study in Ghana demonstrated that the NHIS resulted in increased health-seeking behavior from informal to formal providers. OOP costs were significantly reduced and awareness of the various diseases motivated the adoption of preventive healthcare measures (40).

Appropriate healthcare utilization with CHE reduction was a way of increasing awareness of SHI and effective gatekeeping at primary care with expanded coverage, cost control, and optimization of resource allocation at all levels.

#### Formal and informal sectors

China had three segmented SHI systems that catered to different categories of the population—the formal sector, urban employee basic medical insurance (UEBMI) which was established in 1998, and the informal sector known as urban residents basic medical insurance (URMBI) established in 2007. The premiums of the latter were subsidized by the government. The third SHI scheme was the new cooperative medical system (NCMS), which covered rural residents. The introduction of the URMBI resulted in an unwanted outcome of a significant reduction of 0.94–1.29% offering by some firms' coverage of UEBMI to their formal sector employees, as they inappropriately encouraged their employees to opt for URMBI. Hence, the employees of these firms become underinsured, and the misuse of government subsidies to the URMBI is possible (41).

The presence of a large formal sector was not a guarantee of acceptability of the implementation of an SHI scheme. This was evident in Ethiopia wherein only 32% of 541 government employees had a positive response. Factors influencing the acceptability included self-perceived health status, healthcare service quality, pre-existing medical coverage by employers, and knowledge of SHI. One possible solution was to enhance awareness on the benefits of SHI to the formal sector (42).

The Agrahara scheme was SHI introduced in Sri Lanka in 1997 and was upgraded in 2016. The scheme covered mainly inpatient costs and minimal outpatient costs for public sector employees. An evaluation of the effectiveness of the scheme observed that, for outpatient treatment, the private sector has greater usage than the public sector, resulting in higher CHE rates, whereas, for inpatient care, public facility usage outnumbered that in private, but the usage was only 23%. Overall, the scheme was unsuccessful in reducing CHE, compounded by the fact that family members were not covered by the scheme. Areas for improvement included reduction of costs of outpatient care in terms of medication and diagnostics and enhanced quality of inpatient care for more efficient cost savings. Further, raising the awareness of the benefits of SHI and with better packages would help reduce CHE (43). SHI coverage of the informal sector may be problematic because of premium subscriptions. However, many countries in Latin America had successfully bypassed this obstacle by exercising government budget transfers to cover the informal sector and vulnerable groups in the SHI program (44). SHI implementation was technically feasible with a large formal sector but problematic in situations with a large informal sector.

Successful implementation in both formal and informal sectors must ensure a full understanding of the SHI concept and adequate coverage of both outpatient and inpatient care. Further, protection for the entire family and sufficient funds to be made available must be included as benefits.

#### Strategic purchasing

Strategic purchasing involved three different areas—namely the benefit packages, choice of service providers, and purchase mechanisms, including the types of provider payment andcontracts. In this regard, the SHSDC of the Nepalese government had been advised to consider the experiences of other countries, such as the Philippines, Colombia, and Thailand. SHI implementation should be under the jurisdiction and monitoring of the government but still granted its independence in function. In structure, purchasing systems should guide consumer behavior and ensure quality and efficiency while purchasing services to guide provider behavior. Multiple funds should be integrated into one single pool covering both formal and informal sectors (45). Appointing a third party by an SHI authority as an agency for strategic purchasing may pose severe issues. For example, the agency may fail to develop an effective relationship with the government, consumers (patients), and purchasers, in this case, the SHI body as a principal. The principal must be fully aware of matters related to the agency's internal bureaucracy, administrative capacity, and the ability to achieve the complexity of the tasks (46). An example of such a problem was the appointment of HMOs by NHIS as the agency for the FSSHIP in Nigeria. In particular, NHIS had failed to act in authority and stewardship in the appointment of HMOs as the agency due to this oversight, resulting in the FSSHIP scheme being rather ineffective (47).

Effective strategic purchasing required an institution established by the government, which was granted a certain degree of autonomy but still subjected to government monitoring in terms of governance and transparency. This establishment should be managed by health finance professionals who would comprise the board of directors.

#### Internal migrants

In China, “internal migrants” referred to people who emigrated to another region outside of their registered residency called “Hukou.” Many benefits such as healthcare and other socio-economic services were only accessible to regions where the citizen's Hukou registry was documented. Thus, such migrants would lose their privileges if they relocated to other regions, but their Hukou remained at their original location. This was more acute for rural-urban migrants. This led to problems with on-the-spot settlements of medical bills for them. The proposed solutions required compulsory local enrolments in BMIUE for contractual employees or to encourage others to enroll in BMIUR. Additionally, cross-regional funds for such purposes should be made available (48). Another problem faced by this group is their inappropriate choice of tertiary hospitals for treatment. To avoid unnecessary cost escalation, strengthening the primary care and secondary facilities, coupled with allowing payment portability, was necessary to allow them easier access (49). Further improvements to enable better financial protection for them included the availability of SHI by qualifying migrants for schemes at their new location and improving the portability of SHI schemes (50).

To apply to the Malaysian setting, it would be preferable to have a central federal authority that offered a common national policy but with the distribution of operations at the state levels. This would allow mobility and portability of benefits wherever any internal migration of the populace occurred.

#### Enrolment

Enrolment in SHI schemes could be mandatory or voluntary and could affect the effectiveness of the schemes. This was illustrated in an 8-year longitudinal study of the enrolment pattern of the SHI, known as NHIS in Ghana using the three indicator ratios: coverage, renewal, and growth ratios. The coverage increased from 33% (8.2 million) to about 41% (11.3 million) between 2010 and 2015 and declined to 35% (10.3 million) in 2017. The renewals increased from 44 to 75.4% between 2010 and 2013 and fell to 64% in 2015 before an upturn to 73% in 2017. Specifically, an average annual growth rate of 3% was recorded, which was low. These variations are due to the voluntary nature of the enrolment and renewal policies wherein certain groups of people took advantage of the schemes and entered and exited under circumstances that were favorable for them. There were also differences between the observed administrative regions. Hence, to ensure the effectiveness of the NHIS, mandatory enrolment, as stipulated in the National Health Insurance Act (ACT 852 of 2012), must be enforced (51). A similar finding was noticed in Vietnam wherein response to the SHI only achieved 72% coverage by 2015. Analysis revealed that factors associated with this shortfall were financial burden, sound health status, accessibility of pharmacies, and complicated enrolment processes. The SHI scheme was favored by those with high education and knowledgeable of their benefits and co-payment functions. High-income groups preferred private insurance over SHI. Hence, either mandatory enrolment or offering subsidized premiums should be enforced to ensure acceptance of the SHI scheme (52).

These experiences indicated that effective SHI implementation would require mandatory registration with a structure that disallowed any possibility of attempts to be misused at the convenience of the enrollees.

#### Fund integration

Fragmentation of multiple SHI systems with multi-payer pools potentiated inequity of SHI coverage and benefits, as well as the persistence of CHE. In 2016, in an attempt to reduce the fragmentation of the three existing SHI systems in China, URBMI and NCMS were integrated to form URRBMI. This union resulted in an overall incidence of CHE (15.53%, about 1.10% higher). However, it reduced the intensity of CHE among the poor by 23.38%. This successful CHE reduction could be further enhanced by offering free insurance premiums added with higher reimbursement rates. Co-payments should be minimized and the outpatient benefits package should be extended to include costly disease coverage (53). An obstacle to funding mergers was the issue of acceptance or rejection by various stakeholders. In 2010, the Iranian government enacted a law to integrate all the multiple insurance funds into one single national health insurance institution, except for the Armed Forces fund. However, there was strong opposition from many stakeholders. Some of the reasons included unwillingness to lose their organizational independence, as well as sharing the privileges with others. Portfolio holders were against the loss of their authority and income. Therefore, only the Ministry of Health and Medical Education and a few others were supportive, and it would require more than political will to have seen this through (54).

In a situation wherein merging existing multiple SHI schemes with multi-payer systems is necessary, the integration mechanism would face various obstacles from existing schemes, which would protect their turf. Therefore, having a full engagement with the various stakeholders to compromise and negotiate is crucial to have created a single effective mechanism.

#### Others

a. *Effects of SHI on access and utilization of obstetric health services*.

*Results from HIV*+ *pregnant women in Kenya*. SHI allowed access and higher utilization of obstetric health services for HIV+ pregnant women (55).

b. *Do justice and trust affect the acceptability of Indonesian SHI policy?*

Lay citizens reflected a positive policy acceptability (PA) correlation with trust and evaluation of healthcare services domains of post JKN. Meanwhile, healthcare workers (HCWs) demonstrated positive PA with health systems and institutions, as well as evaluation of healthcare services post JKN. All other measures had no significant correlations in both groups (56).

c. *Decentralization of Indonesian SHI*.

Decentralization of INA-Medicare was acceptable for Aceh province due to the consistency with the Indonesian constitution in implementing regional autonomy and Sharia principles. The move also would lead to quality improvement of public healthcare services (57).

d. *Transnational actors and healthcare reform: Why international organizations initially opposed, but later supported, SHI in Ghana*?

In 2003, the Ghana government demonstrated the political will to implement SHI despite the initial objections and pressures from transnational organizations by using the funds from the heavily indebted poor country (HIPC) allocation. This was followed by the move to decentralize SHI, which accelerated its expansion. By 2005, the SHI policy had successfully implemented nationwide, and subsequently, the transnational actors supported the move (58).

e. *Impact of the COVID-19 Pandemic on SHI Claims for High-Burden Diseases in the Philippines*.

PhilHealth supported the health expenses of 70% of Filipinos, although the financial reimbursement covered between 50 and 60%. The COVID-19 pandemic resulted from a general decline in insurance claims due to the reduction of patients who would have come to seek treatment. Treatment of both acute infectious and chronic non-communicable diseases was reduced. This would lead to severe medical and public health consequences later. Elective surgeries for cases such as cancers too had declined. The pandemic had refrained patients from seeking medical care because of the fear of getting infected and the various lockdowns had restricted their movements. The income loss due to the lockdowns reduced the demand for healthcare because of the lower affordability for co-payments. Additionally, some hospitals had restricted their facilities to cater more to COVID-19 cases (59).

## Discussion

Based on the premise that SHI was a viable option to achieve UHC, many developing countries in Asia (e.g., China and middle-income countries from Africa) had implemented this health finance mechanism. However, studies revealed that its implementation in many of these countries had faced various obstacles at different stages. Although there had been several reports of its successes, many more had found the paths to be fraught with various obstacles. However, such obstacles did not imply total failure as lessons learned had made the identification of possible solutions that resulted in corrective measures in the short term, as well as the redesign of flawed policies or processes for a long-term outlook.

In a rare, clear success outcome related to the coverage of the poor in India, underlying factors were attributed to extensive community outreach efforts with a scheme that provided adequate insurance coverage for high-burden diseases and ease of access to tertiary services for related treatment. SHI had also positively influenced a mindset change from the preference for informal to formal care, including increased preventive health measures. The problem of adequate coverage for informal and underprivileged groups could be alleviated by state budget transfers. Specifically, in the Chinese experience, integrating different existing central government schemes catering to different groups had reduced catastrophic health expenditures. SHI had also enabled increased access to obstetric services. Further, a strong and consistent political will and decentralization of services also contributed to some of the successes achieved.

Meanwhile, many countries faced various challenges at different stages. Some of the most evident included failure to enforce compulsory contributions, ineffective strategic purchasing practices due to agency issues, and inadequate understanding of enrollees on their benefit privileges. Although theoretically implementing SHI would have been easier for the formal sector, areas that required further focus included ensuring high-quality services and providing other concurrent medical coverage. However, more issues are presented with formal sectors. The coverage of both sectors must ensure adequate family coverage in the provision of outpatient care with an emphasis on NCDs and inpatient care that included critical illnesses. Premium payments should consider subsidies and ease of access for the poor, as well as benefits packages that are commensurate. Both public and private providers should be remunerated by fair processes and amounts. Funds also must be flexible for cross-border coverage. Probably one of the most difficult issues would be the integration of multiple pools involving different stakeholders. More recently, the COVID-19 pandemic had created another serious obstacle.

## Conclusion

### Strengths of the study

This study covered the recent 5-year experiences of many countries in pre-defined categories, which were mainly in Asia, Africa, and Latin America. Hence, such observations would be useful for other countries in the same categories to ensure that they would be able to avoid severe pitfalls as well as adopt evidently successful strategies, in their attempts to implement SHI with the hope of achieving UHC.

### Limitations of the study

This study observed the successes and issues of SHI implementation in developing and MICs abstracted from data in the three databases involved, namely PubMed, EBSCO, and Google Scholar. Other relevant data may also be available from other sources, such as Cochrane and Scopus, which were not included. Additionally, comparative studies of the effectiveness of SHI in developed and high-income countries in achieving UHC would be useful as there had been reports of shortfalls experienced in some of these countries (8).

### Recommendations

Despite the recent developments, many of the countries still have gaps, especially in areas related to strategic purchasing, effective involvement of formal and informal sectors, coverage of the poor, and the ultimate achievement of the total UHC.

SHI could be an effective mechanism in health financing. However, to maintain long-term sustainability and full achievement of UHC, it would need to complement together with general taxation.

Despite several successes by some of these countries, more innovations and trials that support by a strong political will for SHI to contribute to an achievement of UHC are still required.

## Data availability statement

The original contributions presented in the study are included in the article/Supplementary material, further inquiries can be directed to the corresponding author/s.

## Author contributions

ANA initiated the concept of the research topic and did the final language proof-reading. ANA, AFAA, and SMA expanded on the concept, design of the study, and revised the first draft of the manuscript critically. MHJ developed the methodology, performed the literature search, selected, assessed and interpreted the articles, and wrote the first draft of the manuscript. SMA recommended the journal for publication. All authors have read and approved the final version of the manuscript.

## Funding

This study was funded by the Faculty of Medicine Fundamental Research Grant, Universiti Kebangsaan Malaysia (Grant ID FF-2020-403).

## Conflict of interest

The authors declare that the research was conducted in the absence of any commercial or financial relationships that could be construed as a potential conflict of interest.

## Publisher's note

All claims expressed in this article are solely those of the authors and do not necessarily represent those of their affiliated organizations, or those of the publisher, the editors and the reviewers. Any product that may be evaluated in this article, or claim that may be made by its manufacturer, is not guaranteed or endorsed by the publisher.
