# Supplementary material for: Successes and obstacles in implementing social health insurance in developing and middle-income countries: A scoping review of 5-year recent literatures
Source: Front Public Health. 2022 Oct 26;10:918188. doi: 10.3389/fpubh.2022.918188 (PMC9648174; doi:10.3389/fpubh.2022.918188)
Supplement: Supplementary file 1 [file Data_Sheet_1.docx]

Supplementary Material

Successes and Obstacles in Implementing Social Health Insurance in Developing and Middle-Income Countries: A Scoping Review of 5-Year Recent Literature

Table 3 Key features of studies

| - Author / year | - Country/location | - Objective(s) | - Type of study | - Population | - Outcome(s) |
| --- | --- | --- | --- | --- | --- |
| 1. Onwujekwe, O. et al., 2019 [14] | - Nigeria | - To evaluate the different health financing mechanisms in Nigeria and the changes required. | - Qualitative | - Public and private sector (government, labor unions, and professional associations) | All four health financing mechanisms underperformed.   - Changes needed to be implemented include compulsory enrollment into FSSHIP including informal sector, focus on strategic purchasing and ensuring beneficiaries’ awareness of their rights. |
| 2. Ogundeji, Y. K .et al, 2019 [15] | - Nigeria | Development of a checklist to ensure a feasible and sustainable SHIS scheme for LMICs.   - (based on concepts; theoretical and empirical evidence). | - Literature Review | - SHIS Officials from the State Ministry of Health and State Primary Health Care Development Agency and representatives from the NHIS Nigeria (Kaduna and Niger) | Final checklist included the following six domains: sources of finance, benefit package, provider payment mechanism, contributing population, and level of compulsion; pooling of funds; administration and management. |
| 3. Okungu, V., et al. 2017 [16] | - Kenya | To assess the financial requirements of both contributory (SHI) and noncontributory (general taxation) to finance UHC in Kenya with a large informal sector. | - Quantitative | - Formal, informal, indigent, and pensioners | Noncontributory (general taxation model) has better long-term financial sustainability, but there is a need for more innovation for general taxation methods. |
| 4. Zhang, Y. et al 2018 [17] | - China | To analyze the crowd-out effect between SHI and private health insurance (PHI) via three channels, namely, savings, demographic factors, and medical expenditure. | - Quantitative |  | Penetration of SHI caused crowd out effect PHI but density of SHI resulted in crowd in of PHI.  Need for efforts to enhance SHI by incentivization through preferential tax policies and control of moral hazards. |
| 5. Obikeze, E., & Onwujekwe, O. 2020 [18] | - Nigeria | Role of HMOs in implementation of the national SHI in Enugu, Nigeria. | - Mixed methods -Partially mixed sequential dominant | - Enrollees of NHIS and HMOs, NHIS manager, providers of healthcare and personnel in the State Ministry of Health | 1. Quantitative study:   Most respondents, including the poor reflected that HMOs did not meet expectations.   1. Qualitative study:   Justification by HMO managers on their roles, but other respondents  rate HMOs very low. |
| 6. Sieverding, M. et al., 2018 [19] | - Ghana and Kenya | To explore private providers’ perceptions of and experiences with participation in two different social health insurance schemes in Sub-Saharan Africa—the National Health Insurance Scheme (NHIS) in Ghana and the National Hospital Insurance Fund (NHIF) in Kenya. | - Qualitative | - Private healthcare providers | Private sector is an important source of healthcare for the poor in many LMICs. Incorporation and accreditation of private sector needs to increase to improve coverage rates among underserved populations.  Private sector concerns also need to be addressed. |
| 7. Miao, Y. et al  2018 [20] | - China | The project aimed to reduce the hospitalization rate among patients  with grade 3 hypertension and improve their  health outcomes, through increasing outpatient service  reimbursement ratio. | - Quantitative | - Patients with Grade 3 hypertension | Increased focus on primary care services could ensure better health outcomes.  Higher SHI reimbursement ratios for outpatient primary need to be prioritized. |
| 8. Le, Q. N. et al 2020 [21] | - Vietnam | To examine:  a. Development of SHI in Vietnam,  b. SHI’s role as financial mechanism towards UHC in terms of population coverage, benefits coverage and financial protection, and  c. Recommendations to achieve UHC. | - Review of government documents | - Society | Five stages of SHI development in Vietnam.  SHI had facilitated progress towards UHC but need to facilitate enrollment for uninsured.  Design of generous benefit packages counterproductive.  Prioritize population coverage and start with small benefit packages that all can access. |
| 9. Shan, L. et al  2017 [22] | - China | To determine SHI performance in the role in UHC and identify challenges in the progress of UHC from viewpoint of insurance managers and administrators. | - Quantitative | - Managers and administrators of SHIs | SHI managers and administrators dissatisfied with achievements of SHI on UHC.  Challenges included limited financial protection, healthcare inequity, poor portability of entitlement, and ineffective supervision and administration of funds.  Need to increase funding capacity investment in health. Ensure consistent policies and efficient fund management. |
| 10. Gu, E., & Page Jarrett, I.  2018 [23] | - China | To address failures of SHI Healthcare reforms in 2009 wherein expenditure only achieved less than 54% of national healthcare expenditure, problem of funds surplus, and unchanged payment methods. | - Narrative | Insurers (purchasers), providers,   - and enrollees under each type of SHI schemes | Changes required to achieve top level SHI structures included the expansion of coverage rate, additional funding, better benefit packages, and reform payment methods. |
| 11. Fenny, A. P. et al 2021 [24] | - Ethiopia, Ghana, Kenya Rwanda, Tanzania | To analyze if SHI is able to efficiently and effectively cover all groups to achieve financial protection. | - Literature Review | Sub-Sharan LMICs >10 M population that have introduced SHI | For countries to achieve UHC with SHI, process must cover all groups right from the beginning and not in sequential phases.  SHI alone in Africa cannot possibly achieve UHC.  Need for combination with tax-based funds to cover all groups |
| 12. Fenny, A. P. et al 2018 [25] | - Ethiopia, Ghana, Kenya, Rwanda and Tanzania, | Comparative study of five African countries on the SHI coverage of the poor. | - Editorial | Sub-Saharan LMICs >10 M population that have introduced SHI | Exclusion of many poor and vulnerable groups from SHI owing to difficulties in identification process despite the legal requirements  Fragmented risk pools caused lower income cross-subsidization among the pools which may result in long term unsustainability |
| 13. El Omari & Karasneh, 2021 [26] | - Philippines | To identify differences in health-related outcomes between indigents with and without SHI coverage. PhilHealth is an  organization managing SHI in Philippines. | - Quantitative | Indigents with PhilHealth and those who did not | Provision of free access by PhilHealth itself was insufficient because the indigents did not benefit.  Need to enhance promotion of community-based care services with free medical supplies.  Emphasize easy-to-understand health promotion and disease prevention efforts. |
| 14Azam, M.  2018. [27] | - India | To evaluate impact of RSBY on RSBY- poor households— Rashtriya Swasthya Bima Yojana, RSBY)—national health insurance scheme (SHI funded by tax revenue). | - Quantitative | RSBY enrollees | No strong evidence that RSBY reduces per person OOP for poor households in both rural and urban areas  RSBY to be replaced with National Health Protection Scheme with increased coverage to 100,000 INR for poor families |
| 15. Sood, N. & Wagner, Z., 2018 [28] | - India | To analyze success of the Vajpayee Arogyashree scheme (VAS), which is a form of SHI that focused on increasing access to tertiary care for households below the poverty line. | Narrative   - (Analysis) | Poor | VAS was successful at effectively and efficiently improving health outcomes.  For patients, the reasons were owing to ease of use, community outreach programs, and focus on high disease burden conditions.  For providers, both public and private practitioners were contracted and remuneration via bundled payments. |
| 16. Liu, K. et al.,  2017 [29] | - China | To investigate the relationships between the MFA (Medical Financial Assistance) grant for SHI enrollment, MFA cash aid and CHE and between SHI enrollment and CHE. | Quantitative | Low-income households | MFA grant has no significant association with low-income households’ SHI enrollment and CHE.  A high SHI enrollment is inversely associated with CHE.  The MFA was ineffective to enhance SHI enrollment and providing financial risk protection for the poor. |
| 17. Ogundeji, Y. K., et al., 2019[30] | - Nigeria | To assess willingness-to-pay( WTP) for SHI in Nigeria. | Quantitative | Households | Positive response to SHI.  82% of households WTP USD 1.68 per person per month/year  Level of acceptance dependent on household size, level of education, occupation and income. |
| 18. Gidey, M. T. et al., 2019 [31] | - Ethiopia | To assess the WTP for SHI and determinant factors  among government servants. | Quantitative and Qualitative (focus group discussion) | Government servants | Higher WTP significantly associated with increasing age and high income.  Lower WTP related with  higher educational status because most respondents were health professionals and felt that they should not pay because their healthcare was already provided for. |
| 19. Akwaowo, C.D et al., 2019 [32] | - Nigeria | - To assess the WTP for a social health insurance among rural residents in the state of Akwa Ibom using double-bound discrete choice analysis. | - Quantitative | - Rural residents in the state of Akwa Ibom | Most respondents (82%) were willing to pay for health  insurance premium. |
| 20. Batbold & Pu   - 2021 [33] | - Mongolia | - To investigate the feasibility of a parallel private health insurance (PHI) alongside the current SHI in Mongolia. | - Quantitative | - Employees from public and private sector firms from different industries. | Mongolian SHI healthcare underfunded with low quality public service.  Need to expand access to SHI package with better benefits and public service quality.   - PHI should be allowed to complement SHI. |
| 21.Thi Thuy Nga, N. et al., 2018 [34] | - Vietnam | - To assess willingness to pay for family-based social health insurance and its determining factors among informal workers in Vietnam. | - Quantitative | - Heads of uninsured households. | A slight majority were not responsive to SHI.  Only 48.8% of 391 respondents were WTP 921.9 thousand Vietnamese dongs per household per year (US$42).  Influencing factors included were household income, number of uninsured members in a household,  and health status of household head.  Actions needed for improvement included affordable premium rates and government subsidies. |
| - 22. Azhar, A. et al., 2018 [35] | - Sarawak, Malaysia | - To assess WTP for SHI (or co-pay) and the associated factors for Sarawak citizens. | - Quantitative | - Citizens from Kuching, Sibu and Limbang | WTP in Sarawak was low.  Agreeable only to amount less than RM 20 per month/year.   - Need to increase awareness on benefits of SHI and to reduce misconceptions so as to raise the WTP. |
| - 23. Tan, S. Y. et al, 2019. [36] | - China | - To analyze impacts of UEI, URI, and NCMS on utilization of health services, total health costs THC, OOP health costs, and mean costs of inpatient and outpatient care. | - Quantitative | - Urban and rural residents in each SHI. | Change from multi-payer to single payer may increase healthcare utilization and costs if SHI benefits are generous.  Cost control measures needed. |
| - 24. Wang, Z. et al, 2018 [37] | - China | - To analyze impact of different types of SHI and other associated factors on healthcare utilization and costs amongst middle-aged and elderly. | - Quantitative | - Middle-aged and elderly enrollees of UEMI, URMI and NCMI. | SHI resulted in increased expenditure on healthcare costs and OOP costs owing to increased utilization.  UEMI caused highest effect.  Associated factors were increasing age and gender.  The three SHI schemes should be adjusted and optimize resource allocation to reduce inequality across the three schemes. |
| 25. Ebunoha, G.N.et al., 2020 [38] | - Nigeria | - To assess the Universal Financial Risk Protection (UFRP) on enrollees and healthcare providers of Formal Sector SHI Program (FSSHIP) in Nigeria. | - Quantitative | - FSSHIP enrollees and healthcare providers | FSSHIP ineffective in providing UFRP and ensuring UHC.  Reduction of OOP by strengthening primary care gatekeeping and expand coverage of FSSHIP, especially inpatient services. |
| 26.Suchman, L. et al., 2020 [39] | - Ghana and Kenya | - To analyze how SHI affects patient decision-making regarding when and where to seek care in Kenya and Ghana. | - Quantitative and Qualitative | - SHI enrollees | SHI raised affordability and health-seeking behavior and potential to expand access to primary healthcare services.  Expand accreditation to private sector owing to lower public sector trust. |
| 27. Abrokwah, et al., 2019 [40] | - Ghana | - To study the relationship between use of formal and informal healthcare with SHI and estimate degree to which formal care substitutes informal care via SHI. | - Quantitative | - Households | SHI enhanced propensity to seek healthcare via preferring formal services over informal ones.  OOP also reduced. |
| 28. Li, X., & Tian, L., 2020 [41] | - China | - To analyze outcome of implementation of a nonemployment-based SHI URMBI (Urban Residents Basic Medical Insurance) on a firm’s decision to offer formal sector UEBMI (Urban Employee Basic Medical Insurance). | - Quantitative | - Formal sector | Crowd-out effect of a non-employment-based program URBMI on an employment-based program UEBMI.  Formal sector workers in UEBMI would be underinsured and government subsidies to URMBI would be misused. |
| 29. Zemene, A. et al., 2020 [42] | - Ethiopia | - To assess the acceptance of government employees on SHI implementation. | - Quantitative | - Government employees | 32% of respondents acceptance suggested poor response.  Factors influencing included self-perceived health status, quality of healthcare service, coverage of medical cost by organization and knowledge of SHI.  Need to improve strategies on benefits of SHI strategies to formal sector employees to increase acceptance. |
| 30. Karunaratna, S. et al., 2019 [43] | - Sri Lanka | To analyze the effect of mandatory SHI   - (Agrahara) on reducing the financial burden of illness on public sector employees in Sri Lanka. | - Quantitative | - Public sector employees in Kalutara district | 1. Outpatient care usage showed private > public sector   Higher CHE rates because SHI only covers inpatient care.  Need to control cost for outpatient care.   1. Inpatient care usage showed   public > private.  SHI not successful in saving from CHE owing to underutilization and family members not covered by SHI. |
| 31.Mathauer, I., & Behrendt, T., 2017 [44] | - Latin America | To identify UHC-conducive institutional design features of government budget transfers for informal sector coverage and assess performance in relation to UHC progress. | - Literature Review | - Informal sector | State budget transfers to health insurance type arrangements is a way to expand coverage of vulnerable groups and informal sector. |
| 32. Sapkota, V. P. & Bhusal, U. P., 2017 [45] | - Nepal | Discuss global experiences and best practices regarding SHI governance for Nepal to learn. | - Viewpoint |  | Social Health Security Development Committee (SHSDC) need to consider independence of function, focus on strategic purchasing, and merge multiple funds into single pool covering both formal and informal sectors.  State supervision of SHI to be enforced. |
| 33. Liu, K., & He, A. J., 2018 [46] | - China | To analyze agency problems in strategic purchasing and agency relationships with government, providers,  and consumers. | - Quantitative | - SHI agencies | Multiple complexity of principal-agent relationships especially on three important areas: purchaser motivation, administrative capacity, and technical complexity of task. |
| 34. Etiaba, E. et al., 2018 [47] | - Nigeria | To assess purchasing arrangements between National Health Insurance Scheme (NHIS), HMOs and healthcare providers; and to determine how NHIS—HMOs—providers arrangements function from a strategic purchasing perspective within the FSSHIP. | - Qualitative | NHIS, HMOs and healthcare providers;   - FSSHIP enrollees | FSSHIP purchasers are not effectively using strategic purchasing tools owing to the dysfunctional two-tiered purchasing mechanism whereby NHIS lacked stewardship in monitoring and guiding the function of HMOs. |
| 35. Yao, Q. et al., 2020 [48] | - China | To analyze the use of health services between internal migrants (IMs) who had local health insurance coverage and those who did not as regards on-the-spot settlements of medical bills. | - Quantitative | - Internal migrants | IMs with local SHI more likely to have health record and use of medical services than those without.  This inequality would further increase health risks in migrants without SHI, especially rural migrants.  Possible rectifications included compulsory local enrollments in BMIUE or in BMIUR.  Cross-regional funds for on-the-spot settlement should be made available. |
| 36. Wang, H. et al., 2018 [49] | - China | To investigate the choices of hospitalization services among internal migrants and the association between SHI and hospitalization choices. | - Quantitative | - Internal migrants | Higher preference for secondary and tertiary hospitals over primary care facilities.  SHI had little influence on choice of facilities.  Need to upgrade primary care facilities to guide patient’s behavior. |
| 37. Chen, W. et al, 2017 [50] | - China | To understand the coverage and financial protection in SHI schemes among rural-to-urban internal migrants (IMs) in China. | - Quantitative | - Rural-urban IMs | Rural-to-urban IMs face difficulties in access to all types of SHI in their current location.  To ensure financial protection, access to SHI and enabling SHI portability, would have to be looked into. |
| 38.Nsiah-Boateng, E., & Aikins, M., 2018 [51] | - Ghana | To examine trends and characteristics of enrollment in the NHIS scheme to inform policy decisions on attainment of UHC. | - Quantitative | - Enrollees of NHIS | Review of last 8 years presented a decreasing trend of enrollment with significant variations among geographical regions and member groups.  Need for enforcement of the mandatory enrollment to increase membership and enlarge the risk pool. |
| 39. Hasegawa, R., 2020 [52] | - Vietnam | To examine the reasons why the Vietnamese do not avail or subscribe to Social Health Insurance (SHI). | - Quantitative | Population from   - 5 provinces—Binh Thuan, Kien Giang, Lam Dong, Nam Dinh, and Phu Tho, and Ho Chi Minh City. | Respondents in sound health did not enroll implied lack of solidarity or adverse selection.  Preference for pharmacies over doctors for medication reflected that problems also arise not only on the demand side but also on the supply side.  Complicated SHI and institutional procedures hindered enrollment.  Need to enforce mandatory enrollment or subsidizing premiums. |
| 40. Wang, J. et al., 2020 [53] | - China | To analyze reduction of inequity of CHE by integration of URBMI and NCMS into URRBMI and assess the determinants of inequity. | - Quantitative | Urban and rural residents under URBMI and NCMS | Integrated URRBMI caused a higher CHE incidence for households, but had some improvement on the intensity of CHE amongst the poor population. |
| - 41. Bazyar, M. et al, 2019 [54] | - Iran | - To observe benefits that the stakeholders may gain or lose in the merger of funds and their acceptance or opposition to the merger. | - Qualitative | - Stakeholders | Successful merger would require considering shareholders’ interests.  Behavior of decision-makers affected by conflicting interests owing to rational choice therapy.  Apply economic approach to rationalize political behavior.   - Strong political will needed. |
| 42. Were, L. P. O. et al., 2020 [55] | - Kenya | To analyze if NHIF increased access to   - obstetric health services for HIV+ pregnant women in Kenya. | - Quantitative | - HIV+ pregnant women (15–49 years | Increase institutional delivery by skilled birth attendants (SBAs) indicated that SHI improved access and utilization of obstetric health services for HIV+ pregnant women. |
| 43. Zein, R. A. et al, 2020 [56] | - Indonesia | To investigate whether justice, trust in healthcare services, confidence level of health system and institutions, political party support, and evaluation of healthcare services (post-JKN) affect policy acceptability (PA) of the healthcare workers (HCWs) and citizens. | - Quantitative | - Citizens minimum 18 years old, healthcare worker (HCW) at least 1 year. | a. Lay people:  PA has positive correlation with Trust and Evaluation of healthcare service post-JKN.  b. HCWs:  PA has positive association with health systems and institutions and evaluation of healthcare services post-JKN.  All other measures have no significant correlations in both groups. |
| - 44. Bintang, S.et al., 2019 [57] | - Indonesia | - Need for decentralization of Indonesian SHI (INA Care) focus on Aceh. | - Review of legal and nonlegal documents |  | Decentralization of INA-Medicare more suitable for Aceh province owing to constitutional provision of regional autonomy, ensuring improvement of public healthcare service quality, and implementation of principles of Sharia SHI. |
| - 45. Wireko, I. & Béland, D., 2017 [58] | - Ghana | - To show the failures of international organization to prevent the implementation of SHI in Ghana. | - Qualitative | - Twenty-two individuals who participated in the development of NHIS | Three factors why Ghana ignored transnational top-down instructions: (a) expensive user fees, (b) to keep their election promises to introduce NHS, and (c) political will of the NPP government that caused the transnational actors to accept the logic of accommodation. |
| 46. Gilbert Ulep, V. et al., 2021 [59] | - Philippines | - To study the effects of the COVID-19 pandemic on PhilHealth inpatient claims for 12 high-burden diseases and five most common procedures in the Philippines. | - Working paper | PhilHealth registrants | Medical claims for 12 high-burden diseases drastically declined by 60% from the previous year.  Acute gastroenteritis, asthma, chronic pulmonary disease, and pneumonia topped the list.  Both public and private hospitals experienced similar levels of decline regardless of the socioeconomic status of the province they are situated in.   - Changes in procedure claims were variable as the lower grade hospitals recorded an increase compared to the highest grades. |

**Abbreviations**

BMIUE (Basic Medical Insurance for Urban Employees), CHE (Catastrophic Health Expenditure), FSSHIP (Formal Sector Health

Insurance Programmes), BMIUR (Basic Medical Insurance for Urban Residents), HMOs (Health Maintenance Organisation), IM (Internal Migrants),

JKN (Jaminan Kesihatan Nasional), LMIC (Lower and Middle-Income Countries), MFA (Medical Financial Assistance),

NHIF (National Hospital Insurance Fund), NCMI/ NCMS (New Cooperative Medical Insurance Scheme), NHIS (National Health

Insurance Scheme), OOP (Out of Pocket), PHI (Private Health Insurance), SHI (Social Health Insurance), SHIS (Social Health Insurance

Schemes), SHSDC (Social Health Security Development Committee), THC (Total Healthcare Costs), UEI/UEMI (Urban Employee

Medical Insurance), UEBMI (Urban Employee Basic Medical Insurance), UFRP (Universal Financial Risk Protection), URI/URMI (Urban Resident Medical Insurance)

URRBMI (Urban and Rural Residents Basic Medical Insurance), UHC (Universal Health Coverage), WTP (Willingness-to-Pay).

**Figure 2 Flow chart of the study selection**

Records identified through database searching PubMed (8,481) EBSCO (36,442) Google Scholar (12,761) (n= 57,684)

Additional records identified through other sources

(Grey literature)

(n=2)

Identification

Screening

Total records identified

(n = 57,686)

Relevant full text articles selected

(n=63)

Records excluded owing to irrelevant titles and abstracts

(n=57,623)

Duplicate articles removed

(n=12)

Studies included

PubMed 18 EBSCO 15 Google Scholar 12 Grey Literature 1

(n=46)

Included

Full text excluded based on irrelevance to research questions

(n=5)

Eligibility

Full-text articles assessed for eligibility

(n=51)

**
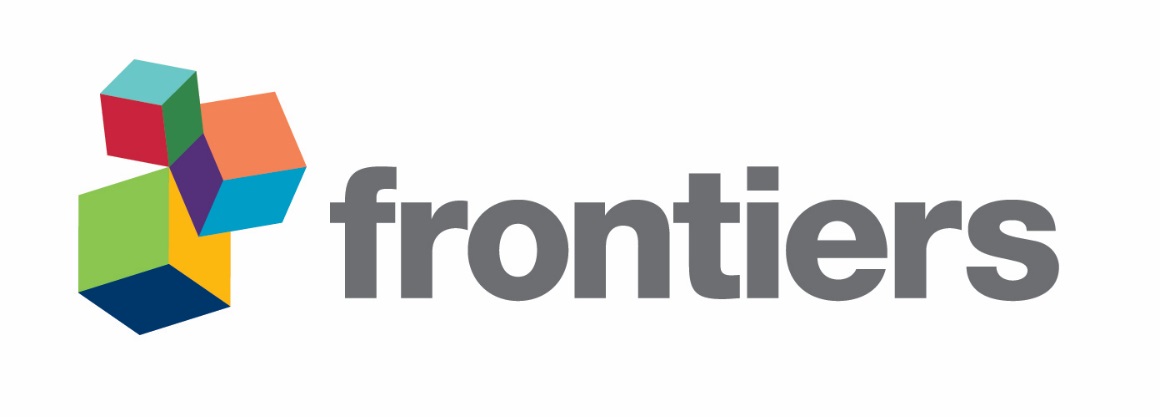
**
